# Hypersensitivity response has negligible impact on Hematopoietic Stem Cells

**DOI:** 10.1016/j.stemcr.2021.06.013

**Published:** 2021-07-22

**Authors:** Nir Bujanover, Roshina Thapa, Oron Goldstein, Leonid Olender, Omri Sharabi, Michael D. Milsom, Roi Gazit

**Affiliations:** 1 The Shraga Segal Department of Microbiology Immunology and Genetics, Faculty of Health Sciences, Ben-Gurion University of the Negev, 84105, Israel; 2 National Institute for Biotechnology in the Negev, 84105, Israel; 3 Center for Regenerative Medicine and Stem Cells, Ben-Gurion University of the Negev, 84105, Israel; 4 Heidelberg Institute for Stem Cell Technology and Experimental Medicine (HI-STEM gGmbH), Division of Experimental Hematology, Deutsches Krebsforschungszentrum (DKFZ) and DKFZ-ZMBH Alliance, 69120 Heidelberg, Germany

**Keywords:** hypersensitivity, immune response, hematopoietic stem cells, dormancy, quiescence

## Abstract

Immune cells are generated from hematopoietic stem cells (HSCs) in the bone marrow (BM). Immune stimulation can rapidly activate HSCs out of their quiescent state to accelerate the generation of immune cells. HSCs’ activation follows various viral or bacterial stimuli, and we sought to investigate the hypersensitivity immune response. Surprisingly, the Ova-induced hypersensitivity peritonitis model finds no significant changes in BM HSCs. HSC markers cKIT, SCA1, CD48, CD150, and the Fgd5-mCherry reporter showed no significant difference from control. Functionally, hypersensitivity did not alter HSCs' potency, as assayed by transplantation. We further characterized the possible impact of hypersensitivity using RNA-sequencing of HSCs, finding minor changes at the transcriptome level. Moreover, hypersensitivity induced no significant change in the proliferative state of HSCs. Therefore, this study suggests that, in contrast to other immune stimuli, hypersensitivity has no impact on HSCs.

## Highlights

1. Hematopoietic stem cells (HSCs) can be activated by viral or bacterial immune stimuli
2. Hypersensitivity stimulation does not change the phenotype of HSCs
3. HSCs’ potency and quiescence are not affected by a hypersensitivity response

## Graphical abstract

**Figure:**
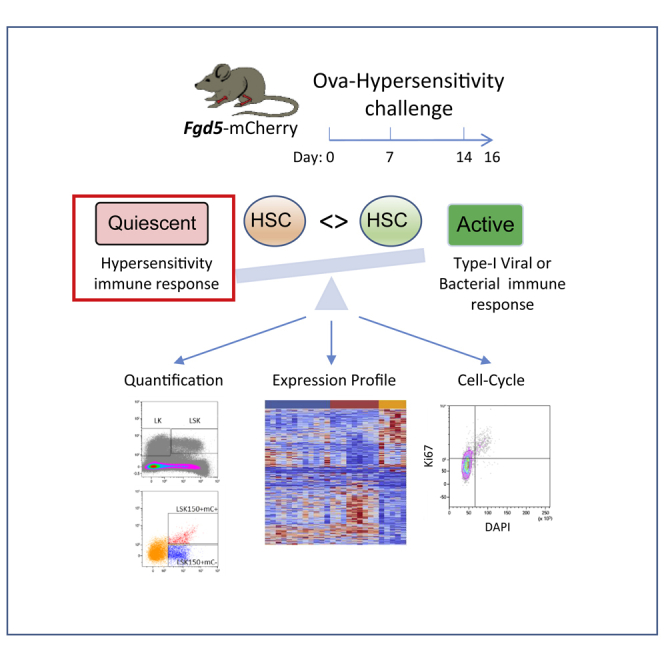

## Teaser

In this article, Bujanover and colleagues show that hematopoietic stem cells (HSCs) are not significantly affected by hypersensitivity immune response. This is surprising, as many other viral or bacterial immune stimuli elicit robust activation of HSCs. The lack of activation may suggest a decision mechanism by which HSCs retain quiescence and potency even through an acute hypersensitivity response.

## Introduction

Immune cells are generated from a rare population of hematopoietic stem cells (HSCs) in the bone marrow (BM) (Dzierzak and Speck, 2008; Gazit et al., 2008; Orkin and Zon, 2008). HSCs are mostly quiescent in the naive BM (Busch et al., 2015; Yamazaki et al., 2007), and slow proliferation is postulated to preserve potency (Bernitz et al., 2016; Chambers et al., 2007; Pang et al., 2017; Sudo et al., 2000; Walter et al., 2015). Studies over the past decade demonstrated induced proliferation of HSCs following immune stimulations, such as poly-inosine-poly-cytidine (pIpC) (Essers et al., 2009; Sato et al., 2009), gamma-interferon (de Bruin et al., 2014), *Mycobacterium* (Baldridge et al., 2010), autoimmune-driven chronic inflammation (Hernandez et al., 2019), or sepsis (Rodriguez et al., 2009). HSC activation may increase the supply of new immune cells needed to fight an invading pathogen. However, prolonged activation may have a deleterious/exhausting impact (Baldridge et al., 2010; de Bruin et al., 2014; Flach et al., 2014; Matatall et al., 2016). Thus, there is great interest in understanding how immune stimulations affect HSCs.

Research of HSCs depends on robust markers (Christensen and Weissman, 2001; Kiel et al., 2005; Osawa et al., 1996; Spangrude et al., 1988). However, several immune stimuli can alter the expression of at least some of the essential HSCs markers, including SCA1 and CD150 (Bujanover et al., 2018; Essers et al., 2009; Haas et al., 2015). We had recently reported the use of the Fgd5-mCherry reporter to identify immune-activated HSCs (Bujanover et al., 2018). This also led to discovering novel markers for HSC activation, including CD317 (Bst2) and CD69. Improved identification of HSCs is crucial for the study of additional immune stimulation.

The immune system polarizes according to the encountered threat. Many studies reported a strong impact of viral or bacterial stimuli on HSCs (Bujanover et al., 2018; Essers et al., 2009; Haas et al., 2015). Hypersensitivity immune response is not well studied with regard to HSCs. BM progenitors and effectors may play a role in lower and upper allergic airway inflammation (Allakhverdi et al., 2009). Eosinophil-derived CCL6 affects HSCs on severe chronic airway inflammation (Zhang et al., 2018). Nevertheless, the role of HSCs remains unclear in the context of hypersensitivity. Moreover, immune responses without pathogens include multiple pathologies in humans (Holgate, 1999), not limited to allergic hypersensitivity (De Luca et al., 2009; Megías et al., 2016). If such immune stimuli might impair HSCs, we had better learn how to minimize damage.

Immune hypersensitivity involves an over-reaction following sensitization and challenge by an antigen or allergen (Tomasiak-Lozowska et al., 2018). Ovalbumin (Ova) is a commonly used allergen in modeling hypersensitivity. Protocols employ a period of sensitization, which exposes animals to the allergen and causes them to develop IgE antibodies, followed by re-exposure to the allergen (activation), usually producing eosinophilia at the site of inflammation (Tomasiak-Lozowska et al., 2018; Zuany-Amorim et al., 1993). Hypersensitivity had been used for studies of so-called T-helper (Th) 2 immune response, modeling human disease (Nakayama et al., 2017). However, the impact of hypersensitivity on HSCs is poorly understood.

Here we study HSCs following hypersensitivity stimulation. Using the Fgd5-mCherry reporter and multiple HSC markers, we found no significant impact of the peritoneal Ova-induced hypersensitivity on HSCs in the BM. Functional examination by transplantation found neither change in potency nor lineage bias of HSCs following hypersensitization. Molecular characterization of highly purified HSCs revealed only negligible gene expression changes. Finally, cell-cycle analysis demonstrated no significant changes in the proliferative status of HSCs, suggesting no major impact of the Ova hypersensitivity on HSCs. This study suggests that stem cell activation by immune stimulation is context dependent and may fit the type of immune response required.

## Results

### The hematopoietic stem and progenitor cells phenotype during hypersensitivity response

**Figure 1:**
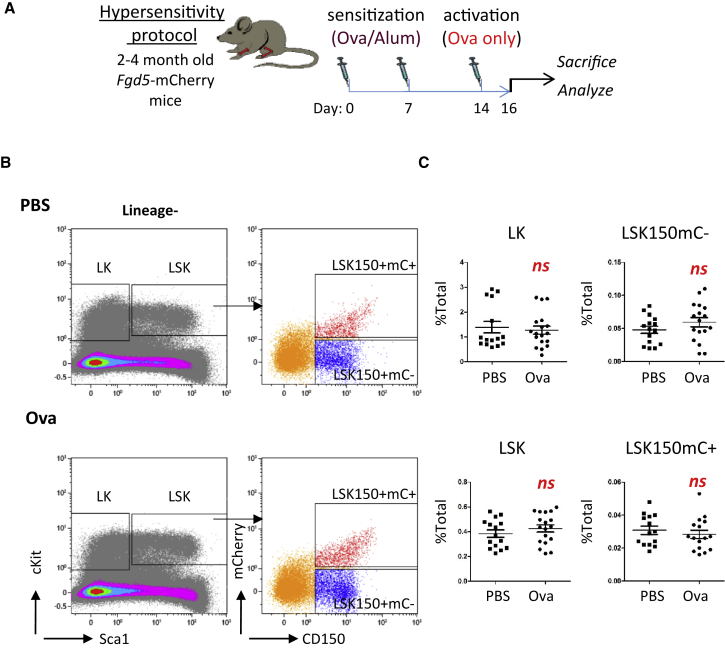
The primitive hematopoietic compartment remains phenotypically normal after hypersensitivity stimulation (A) Workflow: *Fgd5*-mCherry mice sensitized on day 0, 7 with OVA absorbed in aluminum hydroxide (Ova/Alum) and stimulated on day 14 with Ova. Mice were sacrificed on day 16. BM cells isolated and stained (L-lineage cocktail, S-SCA1, K-cKIT, CD150), and gated on four populations: LK, LSK, LSK150, and the *Fgd5-*mCherry^+^. (B and C) Representative flow cytometry plots and quantification. Data are from four experiments n > 3 in each group per experiment. Error bars indicate SD.

We started with sensitization with Ova absorbed in aluminum followed by hypersensitivity activation by the Ova allergen alone (Figure 1A). The proper response was confirmed by observing eosinophils' infiltration into the peritoneal cavity (Figures S1A and S1B). Evaluating this response in multiple time points revealed a peak response at 24–48 h, albeit with substantial variance among individual mice (Figure S1). Importantly, eosinophilia was observed in the peritoneal cavity and the BM, supporting a systemic reaction (Figure S1). We measured the fraction of progenitors that are Lineage^−^cKIT^+^SCA1^−^ (LK), the whole multipotent-compartment Lineage^−^cKIT^+^SCA1^+^ (LSK), and further fractionated Lineage^−^cKIT^+^SCA1^+^CD150^+^Fgd5-mCherry^−^ (LSK150mC−) from the HSCs that are Lineage^−^SCA^+^cKIT^+^CD150^+^*Fgd5*-mCherry^+^ (LSK150mC+, Figures 1B and 1C).

Neither frequencies nor apparent expression of any of the markers tested revealed any significant changes in the hypersensitized mice. Notably, the lack of changes was not observed only in the HSCs compartment (Figure 1C). Thus, hypersensitivity stimulation induced no phenotypic change in the hematopoietic stem or progenitor cells of the BM.

### HSC potency persists following hypersensitivity response

**Figure 2:**
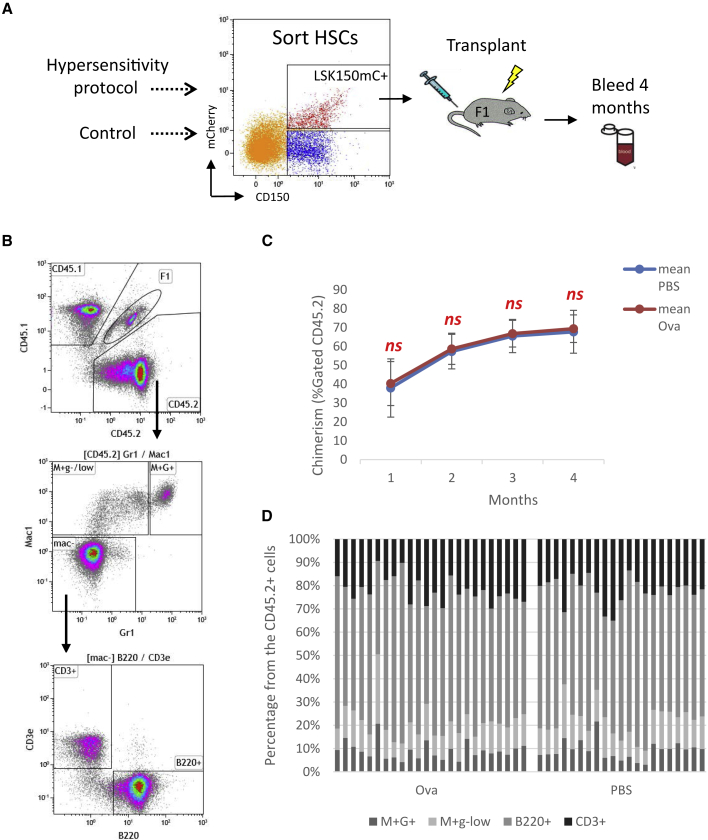
HSCs retain multipotent engraftment potency after Ova-induced hypersensitivity (A) Workflow: donor (CD45.2) hypersensitized as before. BM cells extracted and sorted for Lineage-SCA1^+^cKIT^+^CD150^+^ expressing *Fgd5*-mCherry (LSK150^+^mC^+^). Sorted HSCs were transplanted with BM from competitor (CD45.1) into F1 recipients (CD45.2^+^CD45.1). (B) Gating scheme of PB analysis, engraftment evaluated by the %Gated donor CD45.2 (chimerism). (C) Mean chimerism of each treatment group over time. (D) Multipotency evaluated by dissecting for myeloid (Mac1^+^Gr1^+^, Mac1^+^Gr1^−/low^) and lymphoid (Mac1^−^CD3e^+^, Mac1^−^B220^+^) progeny. Data shown from three experiments, more than two donors in each condition (total for Ova, n = 24; PBS, n = 21). Error bars indicate standard deviation (SD).

We next characterized the function of HSCs from Ova-hypersensitized mice. We transplanted LSK150mC^+^ HSCs from CD45.2 donors that were either control or hypersensitized, along with congenic competitor whole BM (CD45.1), into irradiated recipient F1 (CD45.1+CD45.2, Figures 2A and S2A). We followed the donor cells' engraftment over 4 months. This strategy enabled separation between donor, competitor, and host cells (Figures 2B and S2B).

Neither gross chimerism (%CD45.2^+^ in peripheral blood [PB]) nor percentages of the emerging donor-derived granulocytes (CD45.2^+^Mac1^+^Gr1^+^), other myeloid cells (CD45.2^+^Mac1^+^Gr1^−^), B cells (CD45.2^+^B220^+^), or T cells (CD45.2^+^CD3^+^) showed any significant differences from control. The reconstitution dynamics were similar to hypersensitized donors and controls (Figures 2B and S2C). Thus, we found no changes in HSC potency in terms of the long-term multi-lineage engraftment post hypersensitivity stimulation.

### Molecular characterization of HSCs after hypersensitivity response

**Figure 3:**
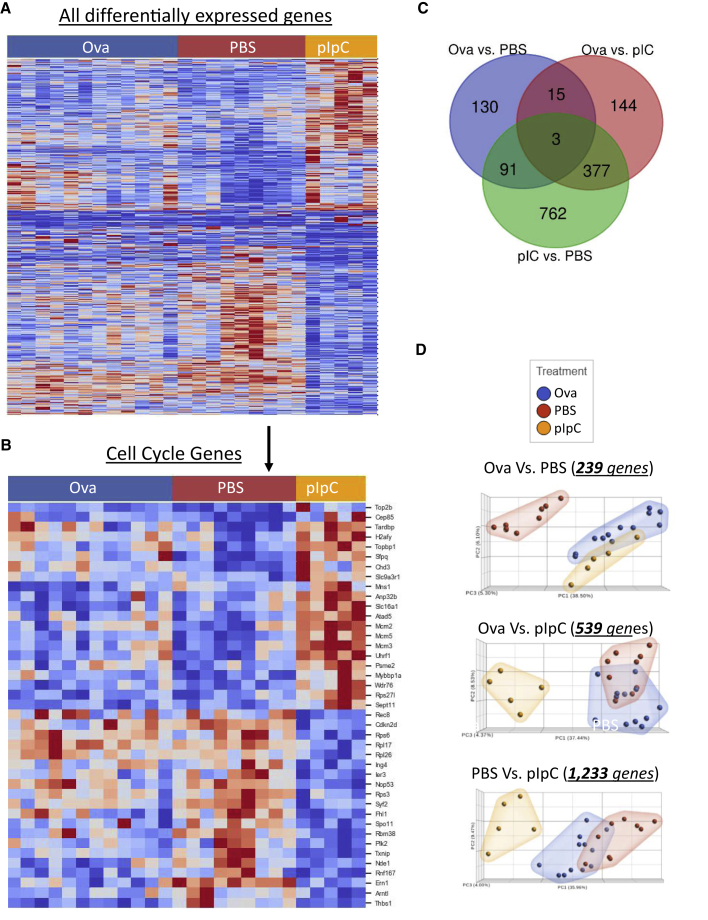
HSC response to hypersensitivity causes only negligible changes in gene expression LSK150mC^+^ cells (HSCs) sorted from control, hypersensitized, or pIpC-treated mice (see Figure 2 legend). DE gene defined if FDR <0.1 and average expression >10. (A) Heatmap of 1,522 DE genes between all groups. (B) Sub-group of cell-cycle genes (GO: 0007049, 41 genes). (C) Venn diagram depicting DE-gene counts between specified groups. (D) PCA of DE genes between specified groups with the number of genes analyzed. Ova, blue; pIpC, orange; and PBS controls, red.

To assess the molecular changes following hypersensitivity, we performed RNA sequencing (RNA-seq) of the LSK150mC+ population from controls, hypersensitized, and pIpC-treated mice to gain the naive, the OVA-sensitized, and the positive control HSCs, respectively. To check for any unique expression signatures, we first evaluated a heatmap of 1,522 differentially expressed (DE) genes (with Average expression >10 and FDR <0.1). Initial observation suggested an excellent clustering of biological replicates (Figure 3A), with possible differences between the three groups. Following the rationale of the positive control group, pIpC showed an expression profile clearly diverging from the control and Ova-treated groups (Figure 3A)

Searching for enriched GO-annotated categories, we did not find significant pathways or groups of genes of interest. Therefore, we analyzed for specific gene groups of interest, such as cell-cycle genes (Figure 3B), HSC-associated genes, cell-surface markers, and inflammatory response genes (Figure S3). The heatmaps suggest that Ova-hypersensitized HSCs have only minor differences from naive counterparts, while pIpC-treated HSCs exhibit pronounced differences. Among the manually examined gene groups, slight changes were observed only in genes associated with the cell cycle.

Another comparison used a simple count, as presented by DE genes, and only a minority corresponded to a unique difference between the Ova-hypersensitized and control. We counted 130 unique/239 total DE genes between control and OVA, in contrast to 762 unique/1,233 total DE genes between control and pIpC. This highlights the similarities of control and Ova-hypersensitized HSCs, and their difference from the pIpC group. Principal component analysis (PCA) on the DE genes further revealed their relations. Relative proximity of the hypersensitivity samples to controls and not to pIpC-treated samples suggests lack of activation. Focusing on the 239 DE genes between Ova-hypersensitized and control (Figure 3D top) initially suggested some relativity with the pIpC, as their cluster seemed to deviate in the same direction. However, PCA of the 539 DE genes between Ova and pIpC showed the overlap of Ova with control (Figure 3D, middle), while pIpC remained well separated. Finally, plotting the 1,233 DE genes between control and pIpC illustrated that the Ova group shared similarities with control rather than with pIpC (Figure 3D, bottom). Taken together, gene expression analysis, in agreement with the phenotypic and functional assays, suggests little if any impact of hypersensitivity on HSCs.

### HSCs retain dormancy after hypersensitivity response

**Figure 4:**
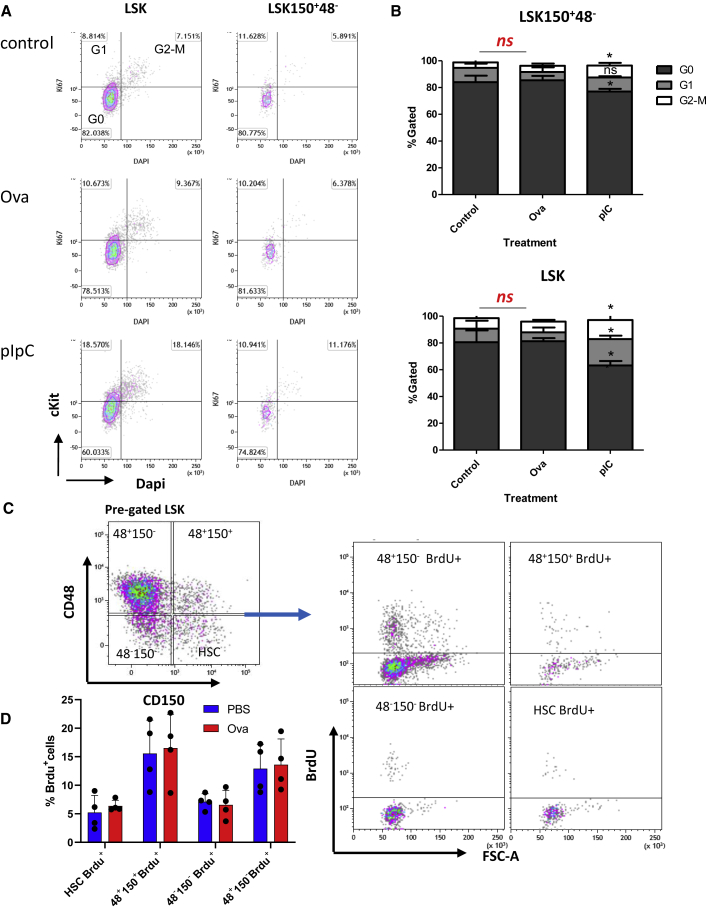
HSCs retain quiescence after hypersensitivity response (A) BM cells from control (PBS), hypersensitized (Ova), or pIpC-treated mice. Representative plots of indicated sub-populations: Lin^−^SCA^+^cKIT^+^ (LSK), LSKCD150^+^CD48^−^ (HSCs). Listed are %Gated of G0 (Ki67^−^DAPI^−^), G1 (Ki67^+^DAPI^−^), and G2-M (Ki67^+^DAPI^+^). (B) Quantification of G0, G1, or G2-M sub-populations. Asterisks indicate significance versus control (one-tail t test: ^∗^p < 0.05, ^∗∗^p < 0.01), n = 3 in each group. Data shown from one out of two independent experiments. (C) BrdU incorporation assay, representative gating plots shown. (D) Quantification of mean %Gated of BrdU^+^ within sub-populations, error bars indicate SD. Data from one of three independent experiments, n ≥ 3 in each experiment. Statistical significance evaluated by two-tailed t test between control versus treatment.

While HSCs are deeply quiescent at naive state (Orkin and Zon, 2008; Pietras, 2017), they can rapidly activate following immune stimulation (Bujanover et al., 2018; de Bruin et al., 2014; Essers et al., 2009, 2011; Haas et al., 2015). Therefore, we performed cell-cycle analysis of BM cells from control phosphate-buffered saline (PBS)-treated, Ova-hypersensitized, and pIpC-treated mice. Staining for Ki67 and DNA content evaluated cell-cycle state: G0, G1, and S-G2-M. We also stained for CD69 and CD317, novel HSC-activation markers that we recently discovered (Bujanover et al., 2018). Figure 4A shows the analysis of defined populations: undifferentiated Lineage^−^, multipotent LSK, and LSK150^+^48^−^ HSCs (since *Fgd5*-mCherry did not sustain fixation). Quantification of cell-cycle states found no significant differences between the Ova-hypersensitized cells and controls, while stimulation with pIpC increased proliferation (Figure 4B). Cell-cycle analysis was further examined using 5-bromo-2′-deoxyuridine (BrdU) incorporation assay, demonstrating that most of the phenotypic HSPCs do not proliferate (Figures 4C and 4D). In spite of variability between individual mice, we observed no significant increase in HSC proliferation following hypersensitivity. On top of that, staining with HSC-activation markers CD69 and CD317 increased robustly following pIpC, but not Ova hypersensitization (Figure S4). Taken together, these data suggest hypersensitization has no impact on HSC quiescence.

## Discussion

Studies on the modulation of HSCs by the immune system have focused on viral or bacterial stimulations, while hypersensitivity has been less studied. Hypersensitivity immune responses are common in humans, with increased frequencies and duration (Julia et al., 2015). If hypersensitivity impairs HSC function, as other immune stimuli do (Baldridge et al., 2010; Haas et al., 2015; Hernandez et al., 2019; Luis et al., 2016; Matatall et al., 2016; Walter et al., 2015), then we have to consider the possibility of a long-term deleterious impact, impaired hematopoiesis, and decrease in potency of numerous immune response mechanisms.

To tackle this question, we used Ova, one of the best-studied hypersensitivity antigens. Ova is a main egg allergen, counting as the second most common food allergen worldwide, affecting 1%–2% of children (Dhanapala et al., 2015). Despite being extremely common, one may avoid a hypersensitivity response by avoiding the antigen; alternatively, oral tolerance can effectively reduce Ova sensitivity (Ramos et al., 2009). Ova-induced hypersensitivity is a standard model that has been in use for many years (Corazza and Kaufmann, 2012; Lambrecht and Hammad, 2015; Zuany-Amorim et al., 1993). Zhang et al. (2018) reported that Ova-induced severe airway inflammation may impair HSCs. In this study, we aimed to understand hematological impact of hypersensitivity in a mouse model of allergic peritonitis. We characterized phenotype, function, gene expression, and cell cycle of HSCs following Ova hypersensitivity. Our data indicate no phenotypic changes in HSCs. The total chimerism and the lineage output of myeloid and lymphoid cells were both similar to unstimulated control (Figure 2). Neither the expression profile nor the cell-cycle state of HSCs changed following Ova hypersensitivity.

While peritoneal eosinophilia ensures that hypersensitization has occurred, the HSCs seem to stay naive, with no detectable changes. Our data suggest multiple DE genes between the Ova-stimulated and control HSCs (Figure 3). However, pathway analysis did not find any significant clusters of genes that we can currently correlate with HSC function. We focused on genes of interest, such as cell cycle (Figures 3B and S3). The number of DE genes is relatively low, especially compared with pIpC stimulation, which counts four times more genes. Therefore, while our data suggest minor changes in gene expression profile, these might be near FDR background noise. Known pathways that are activated by viral or bacterial stimuli may not engage by hypersensitivity. Moreover, using the Fgd5-mCherry reporter (Bujanover et al., 2018) ensures precise identification of HSCs. These findings raise at least two options: either simple inability of hypersensitivity to induce any impact on HSCs or the systemic effect differs in the BM microenvironment whereby the niche protects the HSCs. We may suggest decision-making mechanisms that discriminate between immune stimuli in order to avoid non-essential activation of HSCs.

Previous studies using other stimuli reported significant changes in HSCs' surface markers, most notably SCA1 and CD150 (Baldridge et al., 2010; Essers et al., 2009, 2011; Flach et al., 2014; Haas et al., 2015; Walter et al., 2015). A functional role for SCA1 was reported (Bradfute et al., 2005), while CD150 may not have an essential function in HSCs (Kim et al., 2006) but changes dramatically following stimulation. Recently, the differences between transplantation and endogenous physiological behavior of HSCs was highlighted (Busch et al., 2015), yet it is the transplantation assay that defines the long-term multi-lineage repopulation potency of HSCs. The idea of differential immune activation of HSCs may seem trivial, but to the best of our knowledge has not been demonstrated for hypersensitivity. Moreover, this new idea presents another challenge for the field: identifying the mechanisms underlying the decision making in terms of activation or no activation of HSCs. Various HSC modulators like interferons (Baldridge et al., 2010; Essers et al., 2009; Sato et al., 2009), interleukin (IL)-1 (Hernandez et al., 2019), and tumor necrosis factor (TNF) (Golan et al., 2018; Pronk et al., 2011; Yamashita and Passegue, 2019) have been reported, yet none are unique to the hypersensitivity response. Interestingly, HSCs have low levels of IL-4 receptor and no IL-5 or IL-13 receptors; possible direct and indirect effects of hypersensitivity cytokines will require additional study.

Our study shows that hypersensitivity caused by allergic peritonitis has no significant impact on the phenotype or function of HSCs, suggesting no perturbation to their basal naive state. Therefore, it suggests we may encounter hypersensitivity immune challenges and presumably retain the life-long potency of HSCs, avoiding impairments in hematopoiesis.

## Experimental procedures

### Mice and ethics

All mice were kept in Ben-Gurion University's specific pathogen free (SPF) unit. Mice strains used were *Fgd5*mCherry reporter (Gazit et al., 2014) on C57Bl/6 background (CD45.2); congenic CD45.1 (JAX strain 002014); and F1 hybrids (CD45.1+CD45.2). Mice were 2–4 months old, both female and male, average weight 20–25 g. All experiments were carried out in agreement with the ethical committee guidelines following the Ben-Gurion University and Israel state Institutional Animal Care and Use Committees' approval.

### Immune stimulation

Ova-hypersensitivity protocol adapted from Zuany-Amorim et al. (1993). Mice were injected subcutaneously (SC) on days 0 and 7 with 100 μg of Ova (catalog no. A5503-1G Sigma-Aldrich) dissolved in PBS, absorbed in 0.8–1.6 mg of aluminum hydroxide hydrate by 1 hour shake. On day 14, mice were injected intraperitoneally (IP) with 10 μg of Ova, no adjuvant, and were sacrificed on day 16 (48 h after Ova activation). Control were littermates injected with PBS only. Positive controls pIpC (P1530 Sigma-Aldrich; 200 μg per mouse), as reported (Bujanover et al., 2018).

### Fluorescence-activated cell sorting

BM cells from the tibia, femur, and pelvis were extracted by crushing in cold sample media (PBS with 2 mM EDTA and 2% fetal calf serum). Mononuclear cells isolated over Histopaque (H1083, Sigma-Aldrich) and stained: Lineage, Pacific Blue; SCA1, APC; cKIT, APCCy7; and CD150, PECy7 (BioLegend). PB samples collected in Alsever solution, red blood cell lysis with ammonium-chloride-potassium (ACK), stained: CD45.2, Pacific Blue; CD45.1, APC; CD3e, phycoerythrin (PE); CD11b, PECy7; B220, APCCy7; Ter119, PerCPCy5.5; and Gr1, fluorescein isothiocyanate (FITC) (BioLegend). Fluorescence-activated cell sorting (FACS) Gallios (Beckman-Coulter) and FacsAriaIII (BD bioscience) were used for analysis and sorting. Kaluza analysis software was used to analyze FACS data. Antibodies are listed in Table S1.

### Cell cycle and proliferation analysis

BM cells from the tibia, femur, and pelvis; mononuclear cells enriched over Histopaque and stained: Lineage-Biotin, followed by BV605-streptavidin (two-step protocol); SCA1, APC; cKIT, APCCy7; CD150, PECy7; and CD48, PC5.5. Cells were fixed in 96U plate in 250 μL of 2% paraformaldehyde in PBS at room temperature (RT) for 20 min, washed twice in PBS, permeabilized in 0.25% Tween PBS for 30min at RT, washed twice in 0.1% Tween PBS, stained by Ki67-FITC (1:300 of stock) in 0.1% Tween PBS overnight. DAPI (10 μ g/mL) added before flow cytometric analysis. BrdU-labeled controls, or Ova-hypersensitized mice, injected IP with 3 mg of BrdU (Sigma-Aldrich B5002) at the time of Ova stimulation. After 72 h, BM cells were extracted and stained for LSK, CD48, CD150; fixed and permeabilized (BioLegend catalog no. 424401); DNase treated (Sigma-Aldrich D5025, 0.25 mg/mL in PBS with Ca+2 and Mg+2). BrdU intra-nuclear staining was by FITC-conjugated clone 3D4 (BioLegend catalog no. 364104).

### Transplantation

HSCs were sorted as Lineage^−^SCA1^+^cKIT^+^CD150^+^*Fgd5*mCherry^+^ (LSKCD150^+^mC^+^), and 300 HSCs were mixed with 1.2 × 10^6^ competitor whole BM CD45.1 and injected intravenously into lethally irradiated (900 rad) F1 recipients. PB sampled at 4, 8, 12, and 16 weeks post transplantation by tail bleed.

### RNA-seq

HSCs sorted as Lineage^−^SCA1^+^cKIT^+^CD150^+^*Fgd5*mCherry^+^ (LSKCD150^+^mC^+^) from control (PBS), pIpC (24 h), or hypersensitized (48 h after Ova). Cells were frozen in SMARTer buffer (100 cells per sample, 10.5 μL) and stored at −80°C. Library preparation and sequencing at the Israel National Center for Personalized Medicine (INCPM) facility (Rehovot, Israel), yielding 5 million to 15 million single reads of 61 bases per sample. Data analyzed using Partek (http://www.partek.com) online; briefly, alignment carried out with STAR v2.5.3a (reference index: mm10, Ensembl transcripts release 92), quantification with Partek algorithm (quantify to annotation model; Partek E/M)) followed by normalization and differential-expression analysis DEseq2 v3.5. Annotated MGI (http://www.informatics.jax.org/function.shtml).

### Data and code availability

Raw data and tables GSE133282.

## Author contributions

N.B. and R.G. conceived the study, designed and performed experiments, analyzed data, and wrote the manuscript. N.B. collected most of the data. R.T., O.G., and L.O. collected parts of the data and did some of the experiments. M.M. and O.S. took part in the study design and manuscript preparation.

## Conflict of interests

The authors declare no competing interests.

## Supplemental information

Document S1. Figures S1–S4 and Table S1

Document S2. Article plus supplemental information
