# Supplementary material for: Hypersensitivity response has negligible impact on Hematopoietic Stem Cells
Source: Stem Cell Reports. 2021 Jul 22;16(8):1884–93. doi: 10.1016/j.stemcr.2021.06.013 (PMC8365095; doi:10.1016/j.stemcr.2021.06.013)
Supplement: Document S1. Figures S1–S4 and Table S1 [file mmc1.pdf]

Stem Cell Reports, Volume *16*

Supplemental Information

Hypersensitivity response has negligible impact on Hematopoietic Stem Cells

Nir Bujanover, Roshina Thapa, Oron Goldstein, Leonid Olender, Omri Sharabi, Michael D. Milsom, and Roi Gazit

Supplementary Figure 1

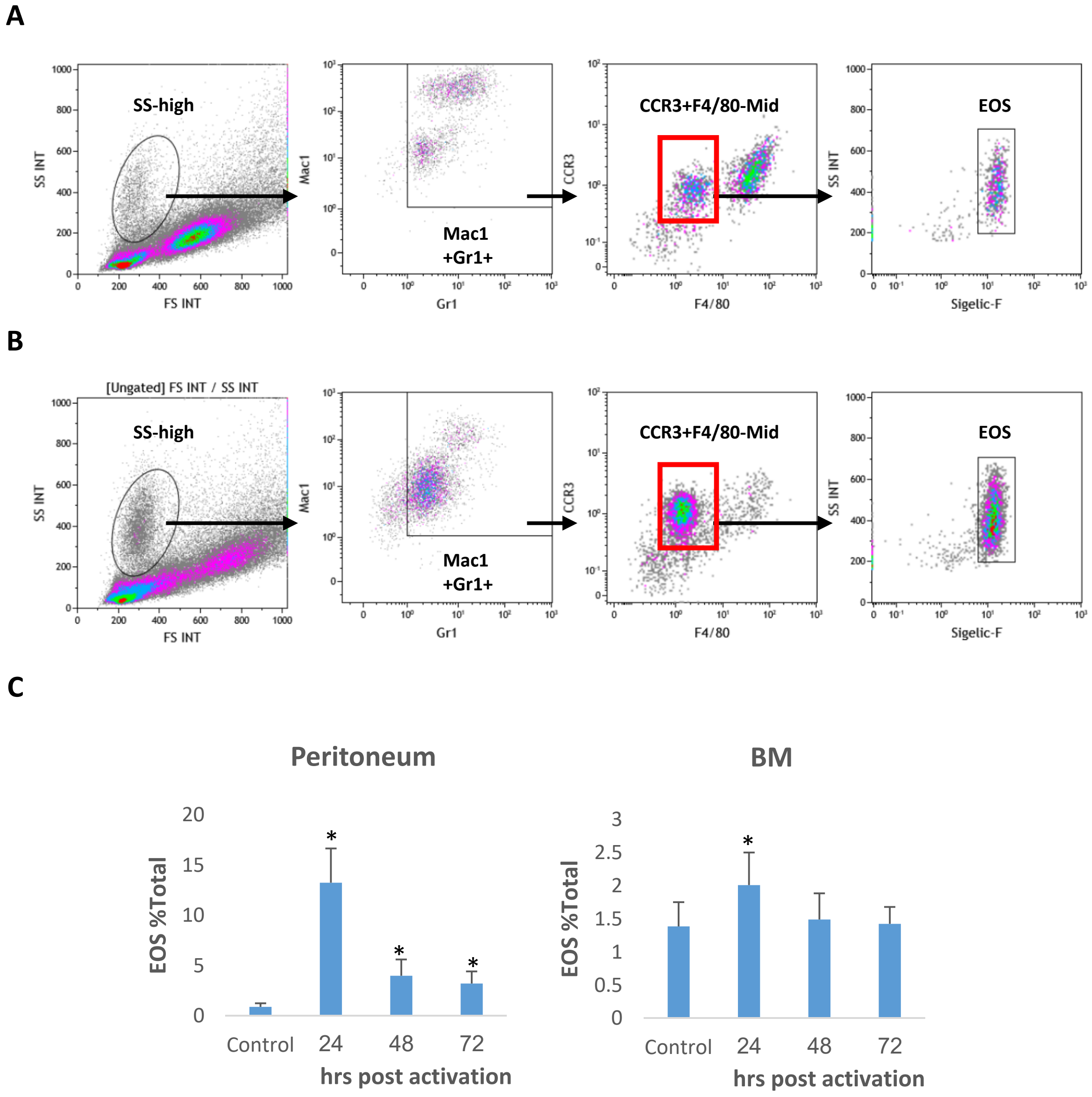

Supplementary Figure 2

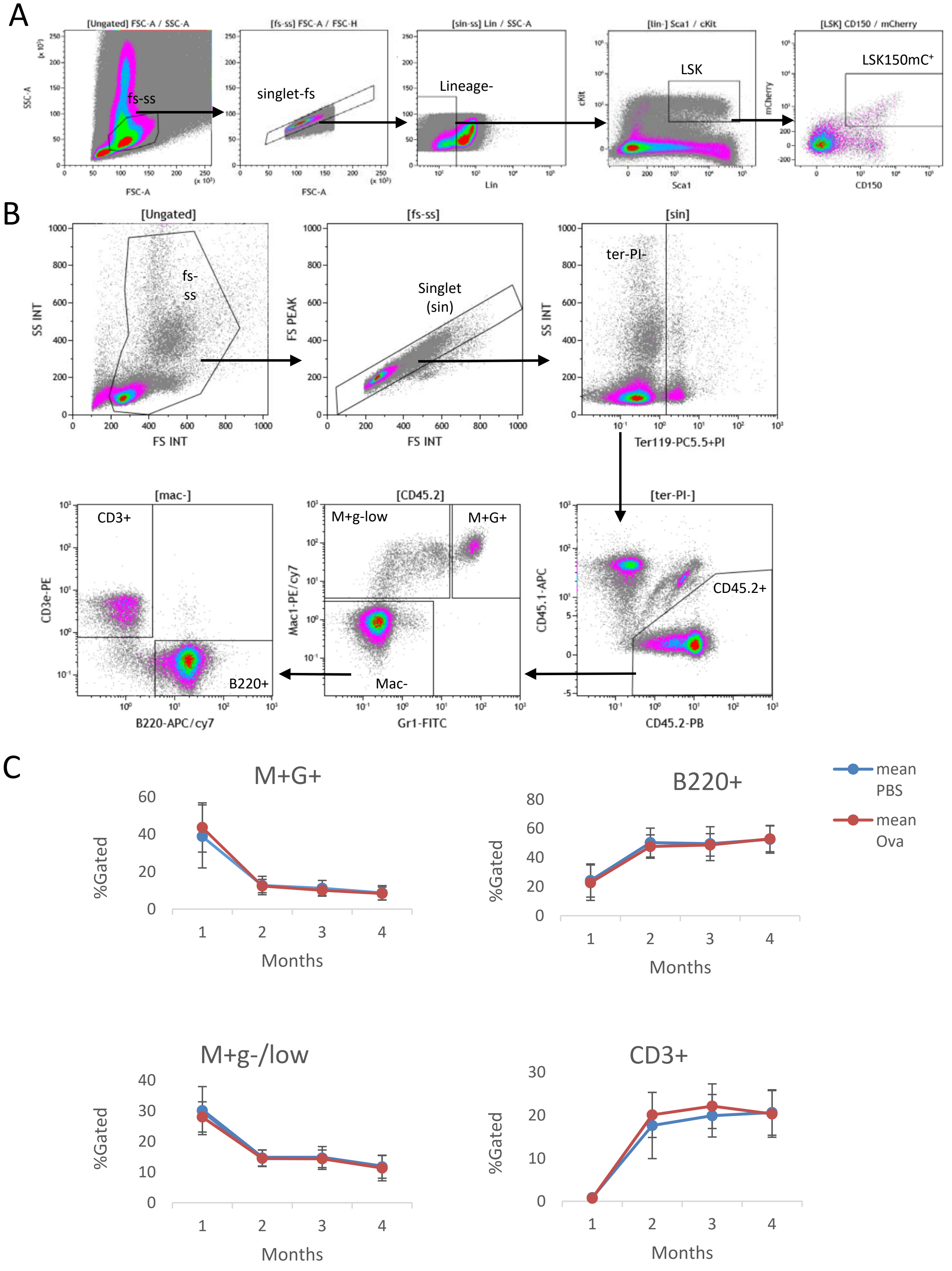

Supplementary Figure 3

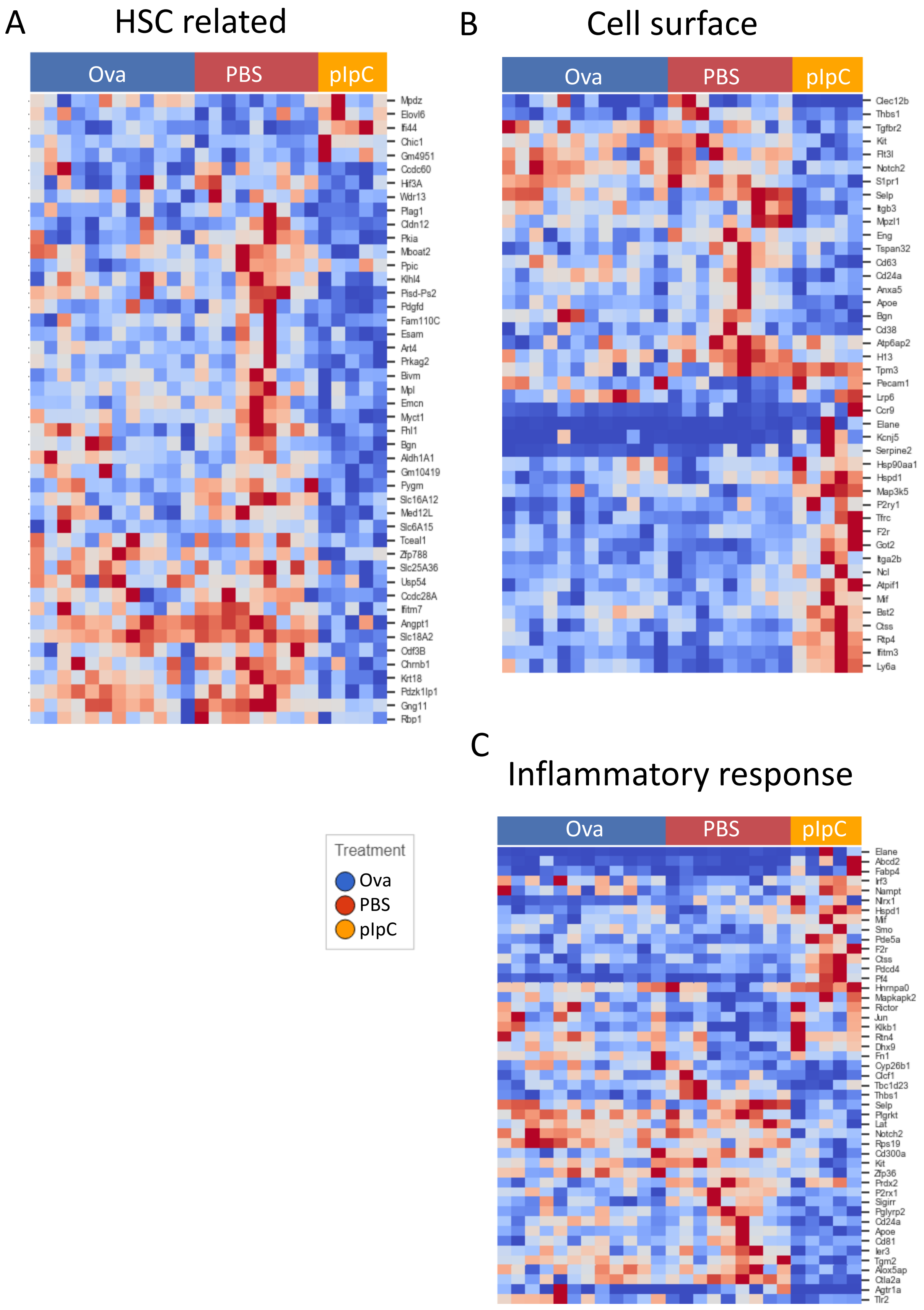

Supplementary Figure 4

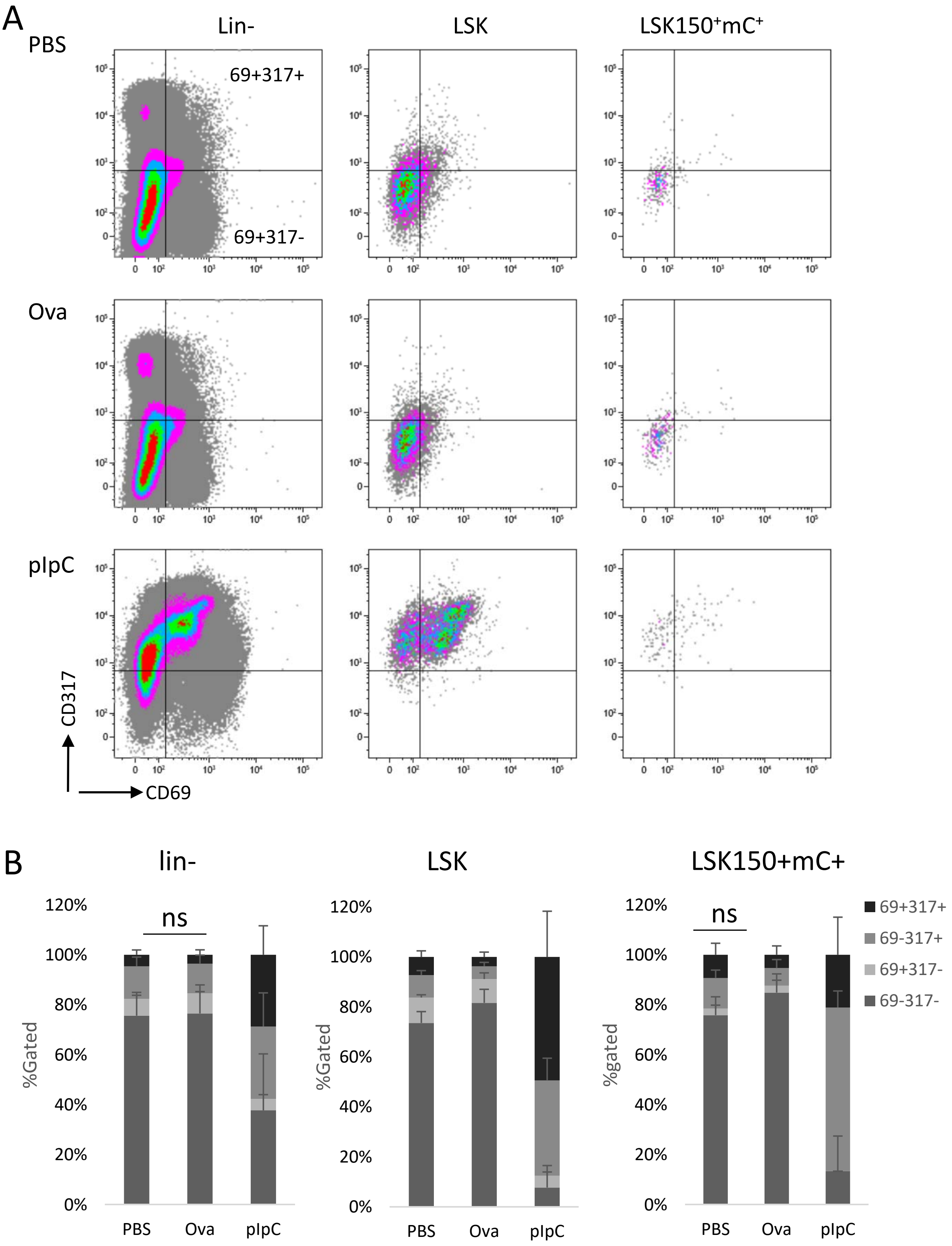

Supplementary Table 1

Anti	Fluorophore	Clone	Lot	Supplier
c-Kit	APC-cy7	2B8	B231077	BioLegend
Sca1	APC	D7	B210898	BioLegend
CD150	PE-cy7	TC15-12F12.2	B210492	BioLegend
CD48	PC5.5	HM48-1	B229042	BioLegend
Lineage cocktial	Biotin-Strep-BV605		B242305	BioLegend
	Pacific-Blue		B223279	BioLegend
Gr1	FITC	RB6-8C5	B138194	BioLegend
Mac1(CD11b)	PE-cy7	M1/70	B249268	BioLegend
B220	APC-cy7	RA3-6B2	B217170	BioLegend
CD3e	PE	145-2C11	C0031122	Tonbo
Ter119	PC5.5	TER-119	B208036	BioLegend
CD45.1	APC	A20	C0453103	Tonbo
CD45.2	Pacific-Blue	104	B169087	BioLegend
Ki67	FITC	16A8	B169139	BioLegend
DAPI			1729803	molecular probs
PI			10p0317	Biotium
CD69	FITC	H1.2F3	B205098	BioLegend
CD317(*Bst2)*	PE	927	B171557	BioLegend
CD201(*EPCR)*	PE	RCR-16	B209944	BioLegend
CCR3	PC5.5	J073E5	B212349	BioLegend
F4/80	APC	BM8	B199093	BioLegend
